# Anchor‐Free Capsular Augmentation Using the Reflected Head of the Rectus Femoris in Hip Arthroscopy

**DOI:** 10.1002/atn2.70312

**Published:** 2026-10-09

**Authors:** Gabriel Octavio Pérez Lloveras, Rodrigo Pérez Dávila, Diego Mengelle, Logan D. Moews, Gonzalo Ezequiel Escobar

**Affiliations:** ^1^ Hospital Universitario Austral Pilar Buenos Aires Argentina; ^2^ Rush University Medical Center Chicago Illinois U.S.A.

## Abstract

Capsular integrity is a key factor in hip stability after arthroscopic procedures. Inadequate capsular closure may contribute to postoperative microinstability, persistent pain, and suboptimal outcomes, particularly in patients undergoing extensive capsulotomy or procedures affecting proximal capsular tissue. Most capsular augmentation techniques using the reflected head of the rectus femoris rely on suture anchors for tendon fixation. This technical note describes an anchor‐free capsular augmentation technique using the reflected head of the rectus femoris. A capsule‐tendon‐capsule suture construct is created using a suture‐passing device, allowing simultaneous capsular closure and reinforcement without additional implants. The technique is simple, reproducible, and cost‐effective and preserves the dynamic stabilizing role of the rectus femoris while maintaining physiologic capsular excursion.

## Graphical abstract

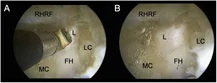

**VIDEO 1:** Anchor‐free capsular augmentation using the reflected head of the rectus femoris in a left hip. The patient is positioned supine on a traction table. Through standard anterolateral and midanterior portals, an outside‐in approach is used to access the joint. After longitudinal capsulotomy and preservation of the reflected head of the rectus femoris, subspine decompression and acetabuloplasty are performed without traction, followed by labral repair under traction. Capsular augmentation is then performed without traction using a capsule‐tendon‐capsule construct. A suture is sequentially passed through the medial capsule, the reflected head of the rectus femoris, and the lateral capsule, and arthroscopic knot tying achieves capsular closure and reinforcement, restoring capsular tension and stability. Video content can be viewed at https://doi.org/10.1002/atn2.70312.

Restoration of capsular integrity has become a critical component of modern hip arthroscopy. The capsule, particularly the iliofemoral ligament, plays a major role in resisting anterior translation and rotational instability.^1^, ^2^, ^3^ Failure to adequately restore the capsule has been associated with persistent pain, microinstability, and inferior clinical outcomes.^4^

Capsular deficiency is especially relevant after procedures involving the proximal capsule, including longitudinal capsulotomy, acetabular rim trimming, subspine decompression, and iliopsoas tenotomy.^5^ In these cases, standard capsular closure (CC) may not provide sufficient tissue coverage or stability.

Augmentation techniques using the reflected head of the rectus femoris (RHRF) have been proposed to reinforce proximal capsular repair.^6^, ^7^ However, most described methods require suture anchors for tendon fixation, increasing implant use and surgical cost. The purpose of this technical note is to describe an anchor‐free capsular augmentation technique using the RHRF through a capsule‐tendon‐capsule construct to reinforce proximal CC.

## SURGICAL TECHNIQUE

### Indications and Contraindications

Capsular augmentation using the RHRF is indicated in patients undergoing hip arthroscopy in whom proximal capsular insufficiency is present or anticipated. Typical indications include longitudinal capsulotomy, acetabular rim trimming, subspine decompression, iliopsoas tenotomy, revision hip arthroscopy, borderline dysplasia, ligamentous laxity, and large capsulotomies. Contraindications include advanced osteoarthritis, irreparable capsular deficiency not amenable to primary closure, and poor tissue quality of the RHRF.

### Anesthesia and Patient Positioning

The procedure is performed under general anesthesia with the patient in the supine position on a traction table. The operative limb is maintained in neutral abduction‐adduction with approximately 10° of flexion, whereas the contralateral limb is positioned in 45° of abduction. Standard sterile preparation and draping are performed.

### Portal Placement and Outside‐In Access

Standard anterolateral and midanterior portals are established under fluoroscopic guidance. An outside‐in approach is used to access the joint through the peripheral compartment prior to traction.

### Capsulotomy and Intra‐articular Procedure

A longitudinal capsulotomy is performed using bipolar radiofrequency, taking the RHRF as the anatomical reference and preserving its insertion. Through this approach, the joint is accessed from the peripheral to the central compartment without traction. The RHRF is identified proximally and maintained intact throughout the procedure to allow its later incorporation into the capsular repair (Figure 1A and B).

**FIGURE 1:**
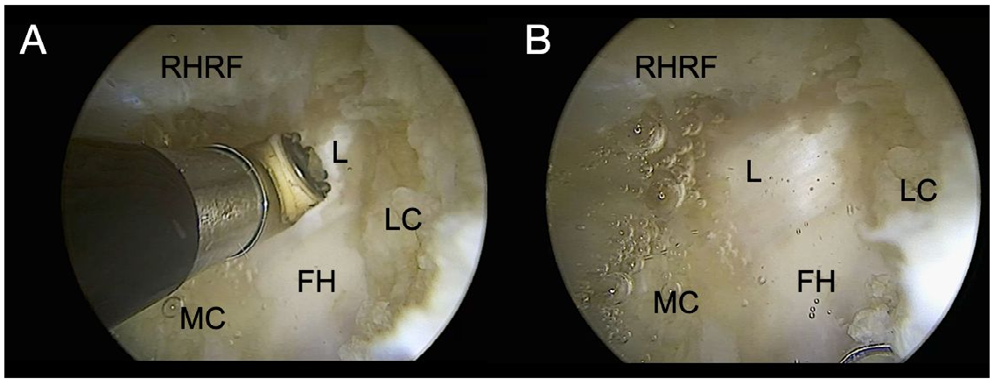
Longitudinal capsulotomy and identification of the RHRF. Left hip, supine position, arthroscopic view from the anterolateral portal with instrumentation through the midanterior portal. (A) Longitudinal capsulotomy is performed using bipolar radiofrequency while preserving the RHRF. The L, MC, LC, and FH are identified. (B) After capsulotomy, the proximal capsule is exposed and the RHRF is clearly visualized for later incorporation into the capsular repair construct. (FH, femoral head; L, labrum; LC, lateral capsule; MC, medial capsule; RHRF, reflected head of the rectus femoris.)

### Central Compartment (Without Traction)

After intra‐articular access is obtained, subspine decompression is performed for a type III anterior inferior iliac spine using a motorized burr. Acetabular rim trimming is then carried out to correct the pincer morphology and achieve balanced acetabular coverage and smooth iliopsoas tracking (Figure 2A).

**FIGURE 2:**
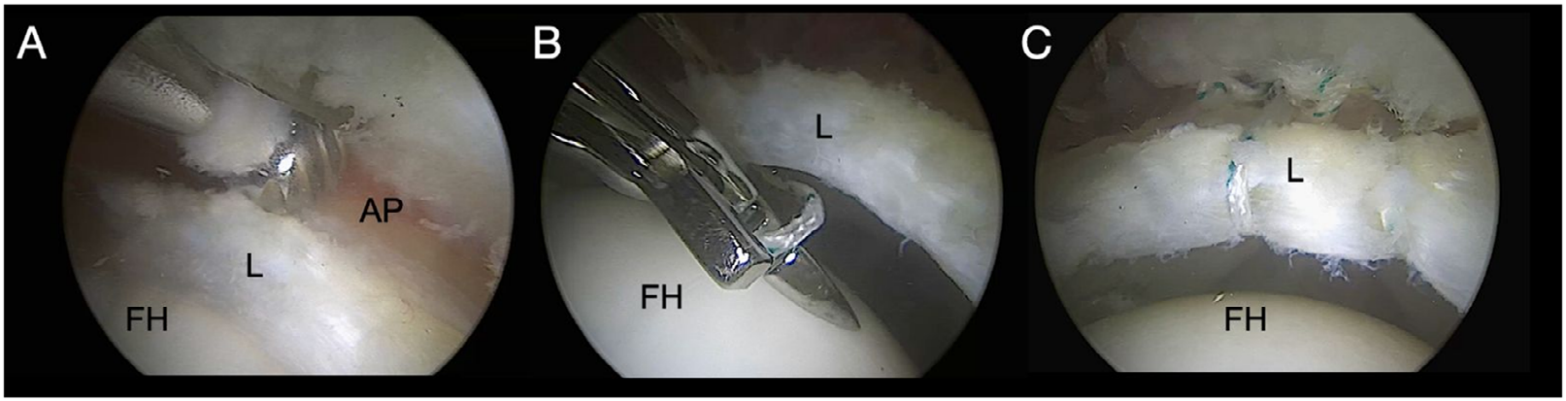
Subspine decompression and labral repair. Left hip, supine position, arthroscopic view from the anterolateral portal with instrumentation through the midanterior portal. (A) AP is performed without traction using a motorized burr to correct pincer morphology and restore acetabular rim contour. (B) Traction is applied to improve visualization of the chondrolabral junction, and sutures from the anchors are retrieved during labral repair. (C) Final arthroscopic image under traction showing anatomic labral repair with anchors in place. (AP, acetabuloplasty; FH, femoral head; L, labrum.)

### Labral Repair (With Traction)

Traction is subsequently applied exclusively to improve visualization of the chondrolabral junction. Arthroscopic evaluation reveals a Konan type II labral tear extending from the 12 to 2 o'clock position. The labrum is stabilized with radiofrequency and repaired using three 3.0‐mm knotless PEEK anchors (Figure 2B and C). Restoration of the suction seal and stability of the repair are confirmed. After completion of labral fixation, traction is completely released. Capsular management is then performed without traction with the hip maintained in neutral rotation and slight flexion.

### Capsular Augmentation Technique (Video Illustration)

Capsular augmentation is performed under direct arthroscopic visualization using a high‐strength nonabsorbable suture loaded onto a FirstPass suture passer (Arthrex, Naples, FL), as shown in Video 1. A capsule‐tendon‐capsule construct is created through the following sequence—(1) medial capsular pass: the suture is passed through the medial capsular leaflet from superior to inferior (Figure 3A and B); (2) first pass through the RHRF: the suture is passed through the RHRF from inferior to superior (Figure 4A and B); (3) second pass through the RHRF and lateral capsular pass: a second bite is taken through the RHRF from superior to inferior to increase tissue capture (Figure 5A and B), after which the suture is passed through the lateral capsular leaflet from inferior to superior (Figure 5C and D); and (4) CC and reinforcement: the suture is tied arthroscopically to approximate both capsular leaflets while incorporating the tendon to achieve CC (Figure 6A and B). An additional side‐to‐side stitch is then placed for capsular reinforcement to ensure a watertight repair.

**FIGURE 3:**
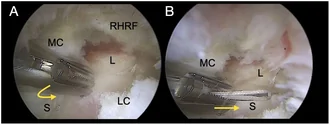
Medial capsular suture passage. Left hip, supine position, arthroscopic view from the anterolateral portal with a FirstPass suture passer introduced through the midanterior portal. (A) The suture passer penetrates the MC from superior to inferior initiating the capsule‐tendon‐capsule construct. (B) The S is retrieved through the capsule completing the first pass. (L, labrum; LC, lateral capsule; MC, medial capsule; RHRF, reflected head of the rectus femoris; S, suture.)

**FIGURE 4:**
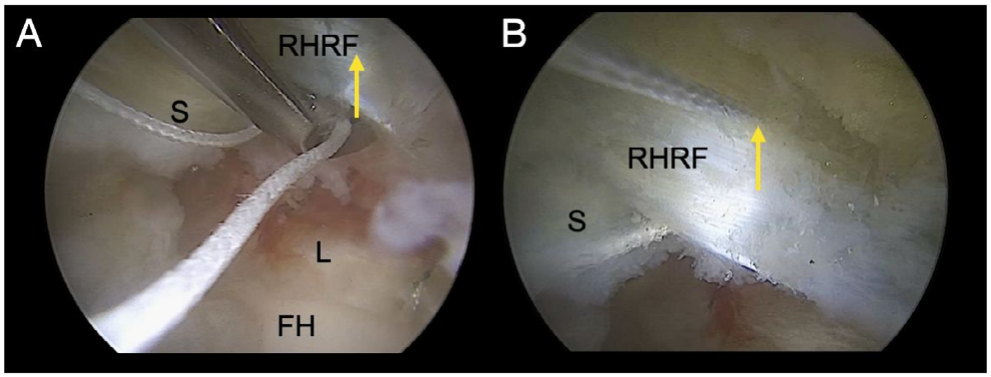
First tendon passage through the RHRF. Left hip, supine position, arthroscopic view from the anterolateral portal with instrumentation through the midanterior portal. (A) The suture passer penetrates the proximal portion of the RHRF. (B) The S is shuttled from inferior to superior through the tendon to initiate biological augmentation. (FH, femoral head; L, labrum; RHRF, reflected head of the rectus femoris; S, suture.)

**FIGURE 5:**
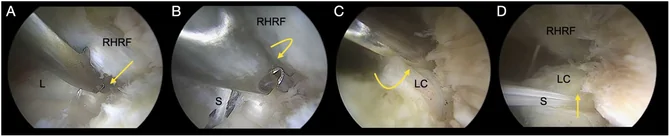
Second tendon pass and lateral capsular passage. Left hip, supine position, arthroscopic view from the anterolateral portal with instrumentation through the midanterior portal. (A,B) A second bite is taken through the RHRF from superior to inferior to increase tissue capture. (C,D) The suture is subsequently passed through the LC from inferior to superior completing the capsule‐tendon‐capsule configuration. (L, labrum; LC, lateral capsule; RHRF, reflected head of the rectus femoris; S, suture.)

**FIGURE 6:**
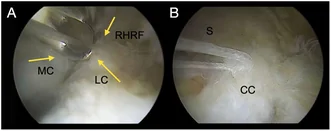
CC and reinforcement. Left hip, supine position, arthroscopic view from the anterolateral portal with instrumentation through the midanterior portal. (A) Arthroscopic knot tying approximates the MC and LC incorporating the RHRF. (B) Final appearance after CC showing a sealed capsule after augmentation. (CC, capsular closure; LC, lateral capsule; MC, medial capsule; RHRF, reflected head of the rectus femoris; S, suture.)

Incorporation of the tendon increases capsular thickness at the proximal leaflet, the area most commonly compromised after subspine decompression and iliopsoas tenotomy. Final probing confirms a watertight closure, and gentle hip range of motion under direct arthroscopic visualization confirms restoration of capsular tension. The sequence of suture passage is illustrated schematically (Figure 7).

**FIGURE 7:**
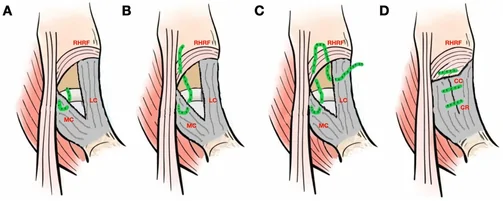
Schematic diagram of the capsule‐tendon‐capsule construct. Diagram illustrating the sequence of suture passage for capsular augmentation. (A) Medial capsular pass from superior to inferior. (B) First passage through the RHRF. (C) Second tendon passage followed by lateral capsular pass completing the construct. (D) Arthroscopic knot tying achieves CC followed by CR. (CC, capsular closure; CR, capsular reinforcement; LC, lateral capsule; MC, medial capsule; RHRF, reflected head of the rectus femoris.)

### Ethics Statement

This study does not involve human subjects research and no patient‐identifiable data are included.

## Postoperative Rehabilitation

Postoperative rehabilitation follows a standardized hip arthroscopy protocol. Partial weight‐bearing with crutches is recommended for the first 2 weeks. Range of motion is progressively advanced while avoiding excessive extension and external rotation during the early postoperative period. Active hip flexor strengthening is avoided for 4 weeks to protect the augmentation construct. Progressive strengthening and sport‐specific activities are gradually introduced thereafter.

## DISCUSSION

The importance of capsular integrity in maintaining hip stability has become increasingly recognized in hip preservation surgery.^1^, ^2^, ^3^ The capsule, particularly the iliofemoral ligament, plays a primary role in resisting anterior translation and rotational forces. Disruption of capsular integrity during hip arthroscopy has been associated with postoperative microinstability, persistent pain, and inferior clinical outcomes.^4^

Procedures involving the proximal capsule, such as subspine decompression and iliopsoas tenotomy, may compromise anterior stabilizing structures despite standard CC.^5^ Capsular insufficiency has been frequently reported in revision hip arthroscopy, emphasizing the need for reliable reinforcement strategies.

Several techniques have been described to address capsular deficiency, including capsular plication and formal capsular reconstruction using grafts or implants.^6^, ^7^, ^8^, ^9^ Although these methods can restore stability, they may require additional implants, graft preparation, and increased surgical time. For this reason, biological augmentation strategies using local tissue have gained interest.

The technique described in this technical note incorporates the RHRF as a local biological reinforcement. Because the tendon originates from the superior acetabular region and is closely related to the anterior capsule, it provides structurally compatible tissue without graft harvesting or fixation to bone.^10^, ^11^ Unlike reconstruction procedures, this approach preserves native capsular continuity while increasing proximal capsular thickness and maintaining physiologic excursion.

An important advantage of this anchor‐free construct is avoidance of implant‐related complications and reduced surgical cost. The technique does not require additional portals or graft preparation and can be reproducibly performed using standard arthroscopic instrumentation (Table 1). It may be particularly useful in patients at increased risk of instability, including those undergoing subspine decompression, iliopsoas tenotomy, revision arthroscopy, borderline dysplasia, ligamentous laxity, or large capsulotomies.

**TABLE 1:** Advantages and Limitations

**Advantages**	**Limitations**
Implant‐free technique	Requires accurate identification of the RHRF
Reduced surgical cost	Risk of overtensioning if excessive force is applied
Preservation of dynamic stabilization	
Simple and reproducible	

RHRF, reflected head of the rectus femoris.

Potential limitations include the need for accurate identification of the RHRF and appropriate tensioning to avoid overconstraint (Table 1). Because the tendon is incorporated as a soft‐tissue reinforcement rather than fixed to bone, the biomechanical strength of the construct relative to other augmentation methods remains unknown and requires further clinical and biomechanical evaluation. Technical pearls and pitfalls of the procedure are summarized in Table 2. Overall, this anchor‐free capsule‐tendon‐capsule construct provides a reproducible biological augmentation option that reinforces proximal CC while preserving normal hip kinematics.

**TABLE 2:** Pearls and Pitfalls

**Pearls**	**Pitfalls**
Clearly identify the RHRF before suture passage	Passing sutures too superficially through the tendon
Maintain moderate tension during knot tying	Excessive tension leading to capsular overconstraint
Ensure adequate visualization of proximal capsular tissue	Inadequate capsular tissue capture

RHRF, reflected head of the rectus femoris.

## DECLARATION OF GENERATIVE AI AND AI‐ASSISTED TECHNOLOGIES IN THE WRITING PROCESS

During the preparation of this work, the authors used ChatGPT (OpenAI, GPT‐5.2) to assist with English language editing and manuscript formatting. The authors reviewed and approved all final content and take full responsibility for the accuracy and integrity of the manuscript.

## DISCLOSURES

The authors (G.O.P.L., R.P.D., D.M., L.D.M., G.E.E.) declare that they have no known competing financial interests or personal relationships that could have appeared to influence the work reported in this article.

## References

[1] Philippon MJ , Trindade CA , Goldsmith MT , et al. Biomechanical assessment of hip capsular repair and reconstruction procedures using a cadaveric model. Arthroscopy. 2012;28:130‐137. 10.1177/0363546517697956 28371596

[2] Abrams GD , Hart MA , Takami K , et al. Biomechanical evaluation of capsulotomy, capsulectomy, and capsular repair on hip stability. Arthroscopy. 2015;31:1511‐1517. 10.1016/j.arthro.2015.02.031 25882176

[3] Wylie JD , Beckmann JT , Maak TG , Aoki SK . Arthroscopic capsular repair for symptomatic hip instability after previous hip arthroscopic surgery. Am J Sports Med. 2016;44:39‐45. 10.1177/0363546515608162 26419897

[4] Domb BG , Philippon MJ , Giordano BD . Arthroscopic capsulotomy, capsular repair, and capsular plication of the hip: Relation to atraumatic instability. Arthroscopy. 2013;29:162‐173. 10.1016/j.arthro.2012.04.057 22901333

[5] Chahla J , Soares E , Devitt BM , et al. Hip capsular deficiency: Diagnosis and management. J Am Acad Orthop Surg. 2016;24:716‐725.

[6] Larson CM , Giveans MR , Stone RM . Arthroscopic capsular plication for hip instability. Arthroscopy. 2011;27:129‐137.

[7] Frank RM , Lee S , Bush‐Joseph CA , Salata MJ , Nho SJ . Improved outcomes after hip arthroscopic surgery in patients undergoing T‐capsulotomy with complete capsular repair. Am J Sports Med. 2014;42:2634‐2642. 10.1177/0363546514548017 25214529

[8] Menge TJ , Chahla J , Soares EA , et al. The role of the hip capsule in arthroscopic hip surgery. J Am Acad Orthop Surg. 2019;27:89‐97.

[9] Mameri ES , Batra AK , Kerzner B , et al. Hip capsular reconstruction with indirect head of the rectus femoris tendon. Arthrosc Tech. 2023;12:e503‐e509. 10.1016/j.eats.2022.12.001 PMC10149979 37138681

[10] Sha SY , Liang T , Liu Y , et al. Proximal capsular augmentation with the indirect head of the rectus femoris for longitudinal capsulotomy in primary hip arthroscopy. Arthrosc Tech. 2024;14:103250. 10.1016/j.eats.2024.103250 PMC11977163 40207347

[11] Philippon MJ , Michalski MP , Campbell KJ , et al. Anatomic relationships of the hip capsule and proximal rectus femoris tendon. Arthroscopy. 2014;30:1628‐1634.

